# London and Counties Medical Protection Society

**Published:** 1916-04-29

**Authors:** 


### LONDON AND COUNTIES MEDICAL PROTECTION SOCIETY.

The Annual Report of the Council of this Society always affords interesting reading, and it includes a record of experiences of which members of the profession would be well advised to take due note. Not even the most prudent and well-intentioned practitioner can be certain that he may not be innocently involved in one or other of the complications which are apt to arise in medical work, and he may learn from the experiences of his colleagues, not only what these complications are but also what are the best ways of meeting them when they arrive. It is remarkable, in view of the dangers which exist, that so many practitioners still remain outside the protection of the societies which are organised for their guidance and defence. The annual subscription is a small one, and the potential benefits are great. Even if the subscriber has to make no appeal for counsel or assistance, the sense of security which results from the consciousness that these are at hand if required is no small advantage. To be covered by the shield of a society powerful alike in experience, in finance, and in prestige, is a comforting reflection, whether actual danger threatens or not, and for his own peace of mind every practitioner ought to avail himself of it. At present far too many practitioners act as though no storm could burst at *their* doors, and they only discover too late that they are no more immune than their neighbours. The situations against which a guard has to be kept cannot always be avoided, seeing that they depend less on the prudence of the practitioner than on the perversity of other people. Patients, or the friends of patients, even when of good faith, are sometimes wrongheaded, and are apt to misinterpret actions which are really for their benefit; or they are misled by inaccurate statements, or are disappointed in their extravagant hopes. Again, a small proportion of people are actively and wilfully malignant, and either to avoid the payment of fees, or from some other motive, they directly attack the character or skill of their medical advisers. Besides these situations in which protection is needed there are problems of action presented by medical practice where even the most cool and clear-headed will be all the better for hearing what an experienced adviser has to suggest. In such circumstances it is an enormous advantage to be able to obtain guidance founded on a large and carefully gathered experience, and to know that the course selected has for its defence a corporate decision and the sanction of those who speak with knowledge. We repeat here—what we have urged in previous years—that it is to the immediate interest of every practitioner to become a member of one or other of the medical protection societies. On the face of things it seems unfortunate that there should be two of these rather than a single organisation. Who, if anyone, is to blame for this division we do not know, and we certainly hold no brief for one society rather than for the other. But we do say that the practitioner who stays outside both of them is running a risk which no sensible man should accept when for a mere minimum of trouble and expense he can securely avoid it.

Still looking at the matter from the point of view of the individual subscriber, it is noteworthy, as the present Report shows, how frequently entanglements or disputes which present a most threatening aspect pass to a rapid and peaceful settlement when the business is put into the hands of experienced negotiators. The practitioner who is subject to some misrepresentation or attack which he knows to be wholly unmerited is very apt, as angry men generally, to reply with more heat than discretion. Easily in these circumstances misunderstandings multiply, and it may be that ere long the doctor finds that in the course of the discussion he has said or written something which has prejudiced his own cause. How different is the course of events when the discussion is from the outset referred to the direction of experienced officials! Mistakes and misunderstandings can much more readily be rectified by the intervention of some individual who has no personal interest in the quarrel, than they can be by the disputants themselves. Again and again the record of the Society's proceedings recites experiences of this order. They are perhaps specially abundant in cases where the patient refuses to pay the fees due to the doctor under the mistaken but honest belief that he has not been properly treated, or that more ought to have been done for him. Such a discussion between doctor and patient may well be difficult to compose by direct negotiation, but, as a matter of fact, is repeatedly composed with good will on both sides when the impartial officials of a medical protection society take the matter in hand. As for the deliberately mischievous and malignant slanderer, he is usually a coward at heart, and while he may attempt to play the bully when he thinks he has only an individual to frighten, his valour usually takes a more chastened form when he learns that an organisation has taken the field. To put the matter broadly, a protection society is an instrument from which the practitioner may get counsel in his difficulties, may be saved from litigation, and may, if litigation cannot be avoided, have his cause efficiently presented to the legal tribunal without the painful arrival of a bill of costs. Let him remain outside these helps and any day, and quite unexpectedly, he may find himself plunged in a dispute which may cause him endless anxiety and annoyance and may involve expenses of no small amount. So much for the narrow personal claim. But societies such as the one here discussed have a wider appeal. If a selected practitioner is not attacked experience shows that some of his *confrères* will be. The incidence of the mischief is unequal, and therefore ought to be met by co-operation and by the principle of insurance. But in order that this position may be secure all those exposed to the risk ought to pay their share. Those who escape are fortunate, and should be cheerful subscribers.

